# Erratum in: Assessment of Neuroprotective Effects of Glutamate Modulation on Glaucoma-Related Retinal Ganglion Cell Apoptosis In Vivo

**DOI:** 10.1167/iovs.66.9.33

**Published:** 2025-07-11

***Erratum in:*** “Assessment of Neuroprotective Effects of Glutamate Modulation on Glaucoma-Related Retinal Ganglion Cell Apoptosis In Vivo” by Li Guo, Thomas E. Salt, Annelie Maass, Vy Luong, Stephen E. Moss, Fred W. Fitzke, and M. Francesca Cordeiro (*Invest Ophthalmol Vis Sci*. 2006;47(2):626-633), https://doi.org/10.1167/iovs.05-0754.

It has come to the authors' attention that the original caption for Figure 2C was incorrect. It used the term SSP as opposed to an OHT (IOP-elevated) model. It also failed to cite the original source of the image. The caption has now been corrected in the article online as follows:

Figure 2. Histologic confirmation that apoptotic cells (**A**, labeled with Annexin V/Alexa-488, *white*) were localized to RGCs (**B**, retrograde labeled with DiAsp4, *gray*), as shown in the combined micrograph (**C**), in an OHT model. **C** is reproduced in part from figure 1B in Ref. 2.

